# Intramedullary injury combined with osteoporosis therapeutics regulates targeted local osteogenesis

**DOI:** 10.1038/s41598-020-80316-y

**Published:** 2021-01-12

**Authors:** Yoko Miyazaki-Asato, Kiyono Koi, Hiroki Fujimoto, Kae Kakura, Hirofumi Kido, Tsukasa Yanagi, Junro Yamashita

**Affiliations:** 1 Department of Oral Rehabilitation, Oral Implantology, Fukuoka Dental College, Fukuoka, Japan; 2 Department of Restorative Dentistry, Oregon Health & Science University School of Dentistry, Portland, OR USA; 3 Department of Oral Rehabilitation, Fukuoka Dental College, Fukuoka, Japan; 4 Center for Regenerative Medicine, Fukuoka Dental College, Sawara-ku Tamura 2-15-1, Fukuoka, 814-0193 Japan

**Keywords:** Preclinical research, Regenerative medicine, Bone

## Abstract

Bone marrow ablation prompts transient bone formation in nearly the entire medullary cavity before marrow regeneration occurs. Here, we establish a procedure to direct bone formation in a desired particular site within the medullary cavity for support of biomedical devices. Local intramedullary injury was performed in the tibiae of rats and parathyroid hormone (PTH), alendronate, or saline was administered. Newly generated bone in the medulla was assessed by micro-CT and histology. To evaluate the function of newly generated bone, animals received intramedullary injury in tibiae followed by daily PTH. At day-14, implants were placed in the endocortical bone and the bone response to the implants was assessed. The fate of newly generated bone was compared with and without implants. We found that neither intramedullary injury nor medication alone resulted in bone formation. However, when combined, substantial bone was generated locally inside the diaphyseal medulla. Newly formed bone disappeared without implant placement but was retained with implants. Bone was especially retained around and between the implants. This study found that local bone marrow disruption followed by PTH or alendronate generated substantial cancellous bone locally in the diaphyseal medulla. This approach offers promise as a tissue engineering tool in medicine and dentistry.

## Introduction

The diaphysis of long bone has a hollow structure filled with predominantly yellow marrow in adults^1^. Trabecular bone is hardly seen in the diaphyseal medullary cavity. When bone anabolic agents or antiresorptives are used, cells in the osteogenic and osteoclastic lineages are stimulated and contribute to improving the bone mineral density in the metaphysis and cortical regions^2,3^. However, those medications are unlikely to induce trabecular bone formation in the diaphyseal medullary cavity even though this region is also located inside the bone. In dentistry, bone regeneration procedures are often performed to augment the alveolar bone for dental implant placement using allograft, xenograft, and alloplast with osteogenic proteins, such as bone morphogenetic protein 2 (BMP2), platelet-derived growth factor (PDGF), and fibroblast growth factor 2 (FGF2)^4^. These procedures induce bone formation on the existing bone surface. In orthopedics, total hip arthroplasty is a predictable procedure but revision replacement is often necessary over time^5^. A major cause of revision replacement is related to implant loosening^6^. Therefore, quality osseointegration is crucial for the long-term survival of hip implants. Bone-anchored prostheses for patients with a lower-limb amputation also require quality osseointegration for long-term stability. Therefore, it is prudent to establish a procedure to build trabecular bone locally in the medullary cavity prior to implant placement. Mechanical bone marrow ablation is one method used to study bone marrow repair^7^. Following bone marrow ablation, the ablated medullary cavity is occupied with reparative trabecular bone and eventually regains bone marrow contents with the disappearance of the reparative trabeculae^8^. To our knowledge, bone marrow ablation is the only method to generate trabecular bone in the diaphyseal marrow cavity. However, it also induces extensive ossification in the cavity in areas where ossification is unwanted. Hence, to develop a procedure to generate trabecular bone in the localized specific desired area in the medullary cavity would be advantageous for implant therapy as well as the treatment of bone degenerative diseases.

Parathyroid hormone (PTH) and alendronate (ALN) are both used for the treatment of osteoporosis. However, their mechanisms of action are different. PTH promotes bone turnover and increases bone mass when administered intermittently^9^, while ALN suppresses bone turnover by inhibiting osteoclasts^10^. We previously reported that intermittent administration of PTH promoted bone formation during osseous wound healing^11,12^ and boosted bone anabolism when administered during growth^13^. Furthermore, intermittent administration of PTH greatly accelerates callus formation during fracture healing in animals^14^. The antiresorptive treatment by ALN has no negative effect on the healing of tibial osteotomies and hip fractures^15–17^. Accumulated evidence also shows that ALN has a positive effect increasing bone mass^18,19^. Since PTH and ALN robustly modulate bone metabolisms, we hypothesize that they would modulate the healing of local bone marrow wounds.

In this study, we, therefore, investigated the effect of PTH and ALN administrations on the healing of local bone marrow wounds to establish a new protocol to build trabecular bone inside the medullary cavity. An animal model was employed since its bone biology provides clinically relevant similarity to human cases. We further studied whether newly formed ossified tissues could be functionally responsive to implants after the cessation of treatment.

## Results

### PTH and ALN administrations for two weeks increased bone mass

**Figure 1:**
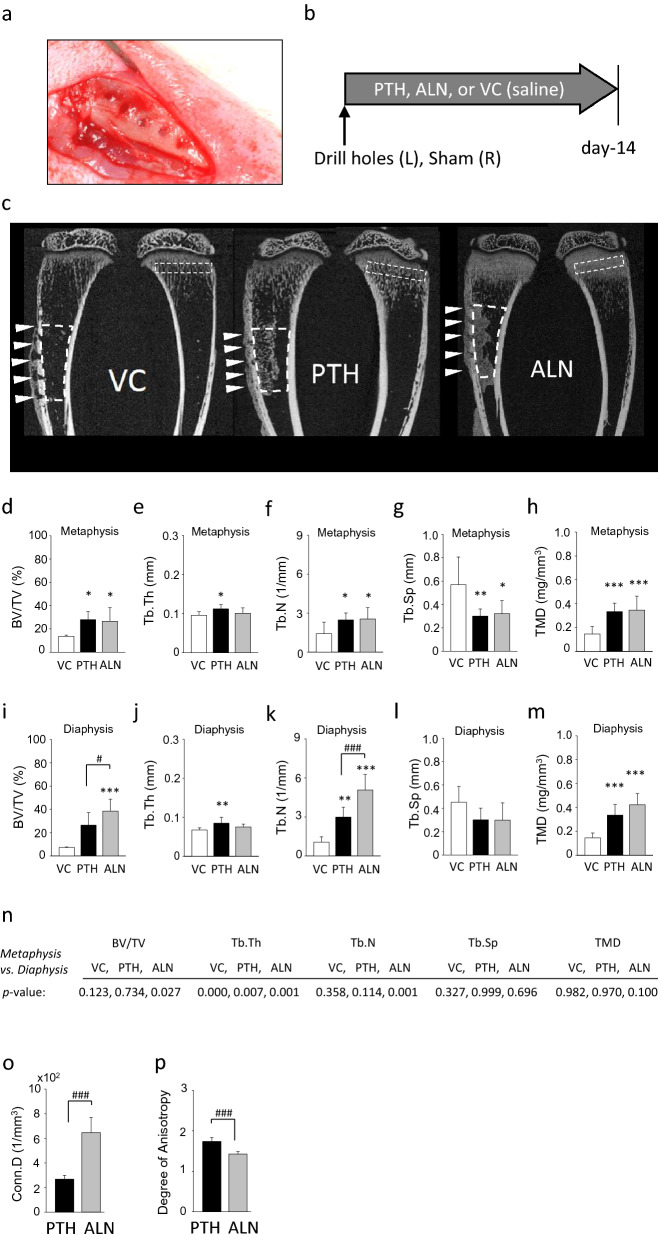
PTH and ALN administrations increased bone mass in rats with substantial trabecular bone formation at the intramedullary injury site. (**a**) An intramedullary injury was created in the diaphysis of the left tibia and sham surgery was performed in the right tibia (n = 33). (**b**) Eleven rats received 20 µg/kg PTH daily; another 11 rats received 0.4 mg/kg ALN twice a week; the remaining 11 rats received VC (saline) for 14 days. (**c**) Representative micro-CT images of tibiae. The trabecular bone within the rectangular boxes in the metaphysis was assessed in the sham tibia to evaluate the skeletal effect of medications. Arrowheads indicate the intramedullary injury sites. Drill holes are not completely repaired in the VC group. Considerable endocortical ossified tissue was noted at the intramedullary injury sites in the PTH and ALN groups. Newly generated bone in the medullary cavity was exclusively found in the intramedullary disturbed tibiae. Minimal trabecular bone was observed in the diaphyseal medullary cavity of the sham tibiae. Since sham sites had very little to no bone to quantify regardless of treatment, the trabecular bone at the local intramedullary injury site was the focus of quantitative analysis. In the metaphysis of the sham tibia, PTH and ALN administration effects were measured via micro-CT analysis showing (**d**) bone volume fraction (BV/TV), (**e**) trabecular thickness (Tb.Th), (**f**) trabecular number (Tb.N), (**g**) trabecular separation (Tb.Sp) and (**h**) tissue mineral density (TMD). In the diaphysis of the intramedullary injured tibia, micro-CT analysis was performed and graphs show (**i**) BV/TV, (**j**) Tb.Th, (**k**) Tb.N, (**l**) Tb.Sp and (**m**) TMD. (**n**) A paired t-test was performed to compare bone parameters between the metaphyseal trabecular bone and the newly generated bone in the diaphysis. *p* values are shown. Connectivity density (**o**) and the degree of anisotropy (**p**) of the diaphyseal trabecular bone was compared between the PTH and ALN groups. All micro-CT data were processed using CTAn software (version 1.16.4.1, http://bruker-microct.com/products/downloads.htm#ctan) which was provided with the purchase of the Bruker X-ray micro-CT system. **p < 0.01; ***p < 0.001 vs. VC, ^#^*p* < 0.05; ^##^*p* < 0.01 between PTH and ALN.

The effect of PTH and ALN administrations for two weeks on the skeleton was assessed in the proximal metaphysis of the sham-operated tibiae (boxed area in Fig. 1c). As anticipated, the PTH and ALN administrations significantly increased bone mass with higher trabecular bone numbers, increased thickness, less separation, and greater tissue mineral density (TMD) when compared to VC (saline) (Fig. 1d–h).

### Endocortical trabecular bone formation by intramedullary injury plus PTH or ALN

**Figure 2:**
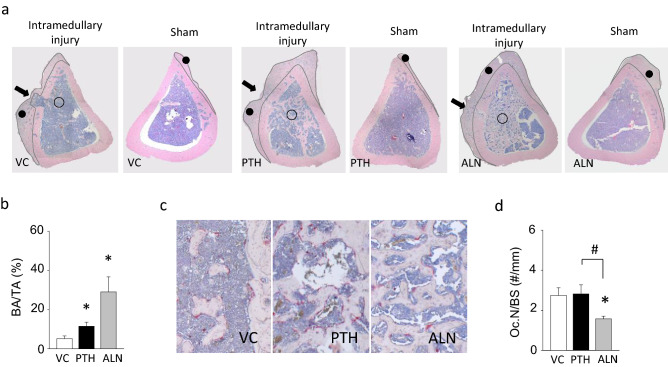
ALN induced greater diaphyseal trabecular bone with suppressed osteoclasts than VC or PTH. (**a**) Representative images of HE-stained sections of tibiae at the injury level. Arrows indicate drilling sites; open circles indicate newly formed ossified tissue; closed circles indicate periosteal ossified tissue. (**b**) The average endosteal bone area at the local intramedullary injury level was significantly larger in the PTH and ALN groups than the VC group. No significant difference was found between the PTH and ALN groups although a trend of the larger bone area in the ALN vs. PTH group was observed. (**c**) Representative images of TRAP-stained sections of the endocortical ossified tissue. (**d**) Osteoclast numbers per bone perimeter were significantly smaller in the ALN group than the VC or PTH group. No differences were noted between the PTH and VC groups. *p < 0.05 vs. VC, ^#^*p* < 0.05 between PTH and ALN.

Bone marrow injury by drilling followed by PTH or ALN administration resulted in generation of substantial bone in the medullary cavity, while local intramedullary injury plus VC administration resulted in little bone generation at day-14 (Fig. 1c,i–m). This newly generated bone induced by intramedullary injury plus PTH or ALN showed a thinner trabecular structure compared to that seen in the respective metaphysis (Fig. 1n). When the outcomes of PTH and ALN were compared, ALN exerted significantly greater effectiveness resulting in increased bone volume fraction, trabecular number, and trabecular bone connectivity density (Fig. 1i–o). The degree of anisotropy (DA) of the newly generated bone in the diaphysis was different between the PTH and ALN groups (Fig. 1p). The diaphyseal trabecular bone in the ALN group resulted in a significantly less anisotropic structure compared to that in the PTH group. On the other hand, minimal bone formation was observed in the medullary cavity without intramedullary injury, irrespective of the treatment. These micro-CT findings were further confirmed in the HE-stained histological sections of the tibial shafts (Fig. 2a). Local intramedullary injury plus PTH or ALN significantly increased bone in the medullary cavity compared to VC (Fig. 2b). Although not significant, ALN increased bone area more than PTH. Intramedullary injury in the VC group resulted in slight generation of trabecula bone in the diaphyseal cavity compared to sham. When PTH and ALN were administered, the osteogenic effect was substantially enhanced. No differences were found in the numbers of osteoclasts per bone perimeter between the PTH and VC groups (Fig. 2c,d). However, the osteoclast numbers were significantly smaller with the ALN administration vs. both VC and PTH.

Periosteal bone formation was large in the intramedullary injured bone and minimal on the sham side (Fig. 2a). This indicates, regardless of treatment, that intramedullary injury promoted periosteal ossification. However, considerably more periosteal ossification took place when PTH or ALN was administered following local intramedullary injury. Therefore, PTH and ALN promoted outer periosteal bone formation as well inside the diaphyseal cavity resulting in even more bone available for anchorage.

### Elevated serum osteocalcin levels by PTH but not by ALN

**Figure 3:**
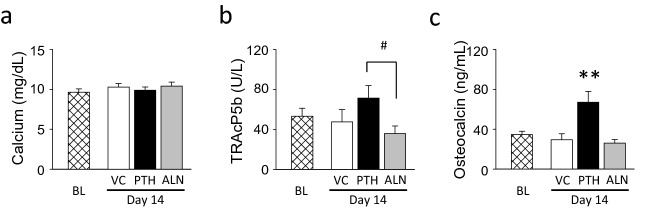
Serum biochemistry. (**a**) Serum calcium levels were maintained within the normal range regardless of treatment. (**b**) No statistical differences were noted in the serum TRAcP5b levels in all treatment groups relative to BL. There was a significant difference at day-14 between the PTH and ALN groups. (**c**) Serum osteocalcin levels were significantly elevated in the PTH group at day-14 in comparison to BL and the other groups at day-14. No difference was noted between the BL and day-14 ALN or VC groups. **p < 0.01 vs. BL, ^#^*p* < 0.05 PTH vs. ALN.

Serum calcium levels were not affected by procedures (Fig. 3a). No significant difference in the serum TRAcP5b level was found between BL and day-14 in any groups (Fig. 3b). However, at day-14, TRAcP5b levels tended to be higher in PTH and lower in ALN compared with VC. There was a significant difference in the day-14 TRAcP5b levels between PTH and ALN. Serum osteocalcin levels, which are associated with bone formation, were significantly elevated at day-14 in the PTH group (Fig. 3c). ALN treatment appeared to have no effect on the serum osteocalcin level.

### Osteogenic gene regulation

The effect of intramedullary injury with or without PTH or ALN administration on the osteogenic gene expression in the diaphyseal bone marrow was assessed at the RNA level using a PCR array. Table 1 shows the comparisons between VC and either PTH or ALN in the sham bone marrow (left column), the mechanically disturbed bone marrow (middle column), and between sham vs. intramedullary injury within each treatment group (right column(s)). Upregulated genes were included with a fold change of 1.5 and above and downregulated genes were included with a fold change of 0.5 and below. For the effect of PTH and ALN on sham bone PTH significantly upregulated matrix metalloproteinase-2 (Mmp2), cartilage oligomeric matrix protein (Comp), bone morphogenetic protein 5 (Bmp5), and integrin alpha 2 (Itga2), while ALN significantly upregulated Mmp2 only compared to VC (Table 1 Left). Both PTH and ALN significantly downregulated collagen type II alpha 1 chain (Col2a1) and cathepsin K (Ctsk). Interestingly, when assessing the effect of local intramedullary injury plus PTH or ALN on osteogenic gene expression in the diaphyseal bone marrow, no osteogenic genes were upregulated by PTH or ALN compared with VC (Table 1 Middle). While ALN treatment significantly suppressed Col2a1 and Ctsk, no osteogenic genes were significantly downregulated by PTH in the mechanically disturbed bone marrow. However, a trend was observed that Ctsk and Col2a1 were downregulated by PTH. Osteogenic gene expression was compared with and without local intramedullary injury in each treatment group (Table 1 Right). No significant differences were noted in osteogenic gene expression with and without local intramedullary injury in the VC and PTH groups. In the ALN group, local intramedullary injury induced the significant upregulation of SMAD family member 5 (Smad5). Although statistical differences were not reached, there was a strong trend that local intramedullary injury upregulated Bmp3 in all groups.

**Table 1:** Summary of up- or down-regulated osteogenic genes in the diaphyseal bone marrow.

(R) Sham				(L) Intramedullary injury				VC		PTH		ALN	
PTH vs. VC		ALN vs. VC		PTH vs. VC		ALN vs. VC		L vs. R		L vs. R		L vs. R	
Fold change		Fold change		Fold change		Fold change		Fold change		Fold change		Fold change	
Bmp5*	2.36	Mmp2*	2.20					*Bmp3*	2.91	*Col4a1*	5.31	*Bmp3*	3.25
Mmp2*	2.30	*Gdf10*	2.03					*Sost*	2.26	*Csf3*	2.55	*Col1a2*	2.46
*Gdf10*	2.17							*Col4a1*	2.19	*Col2a1*	2.38	*Mmp10*	2.03
*Col6a1*	2.04							*Col1a2*	1.94	*Bmp3*	2.23	*Ctsk*	1.97
Comp**	2.03							*Col1a1*	1.85	*Ctsk*	1.92	*Bmp7*	1.94
*Col1a2*	1.98											Smad5*	1.93
Itga2**	1.89												
*Fgf1*	0.53	*Fgf1*	0.53	*Fgf1*	0.40	*Phex*	0.35			*Gdf10*	0.39	*Fgf1*	0.49
*Col4a1*	0.42	*Phex*	0.35	*Ctsk*	0.31	*Bmp3*	0.35						
Ctsk**	0.25	*Bmp3*	0.31	*Col2a1*	0.24	Ctsk**	0.20						
Col2a1**	0.11	Ctsk**	0.16			*Fgf1*	0.18						
		Col2a1**	0.15			Col2a1**	0.17						

Upregulated genes with a fold change of 1.5 and above and downregulated genes with a fold change of 0.5 and below are shown.

Statistical difference between groups, *: *p* < 0.05, **: *p* < 0.01.

### Implants maintained newly formed bone

**Figure 4:**
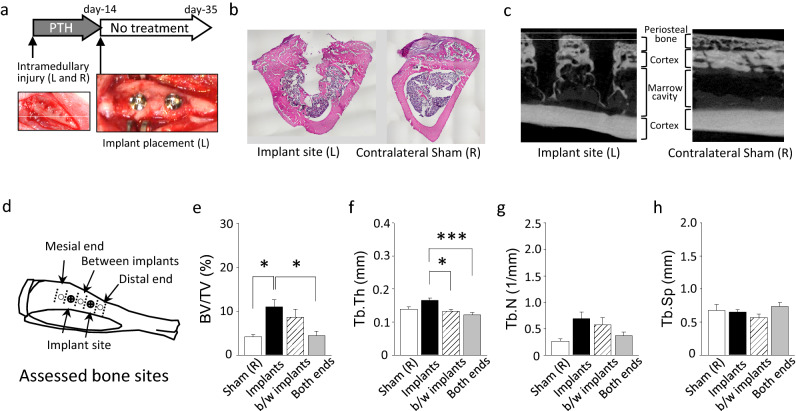
Implant placement contributed to the survival of the diaphyseal trabecular bone. (**a**) Seven rats received intramedullary injury in both tibiae, followed by PTH administration for 14 days. After the completion of treatment, screw implants were placed in the left tibia and sham surgery was performed on the right tibia. Rats were euthanized 3 weeks after implant placement and survival of trabecular bone in the medullary cavity was assessed. (**b**) Representative images of HE-stained sections and (**c**) micro-CT images of tibiae at the injury level. A significant amount of trabecular bone was retained in the medullary cavity at implant sites, while minimum trabecular bone was observed in sham sites. Considerable periosteal bone was retained in both implant and sham sites. (**d**) The cartoon indicates the bone segments assessed. Micro-CT analysis results depicted in graphs showing the bone volume fraction (**e**) was higher in the bone around implants than the bone at the mesial and distal ends. (**f**) Trabecular thickness was larger in the bone around implants than in the bone between implants or at the mesial and distal ends. The endocortical trabecular number (**g**) and separation (**h**) were similar between the bone segments. All micro-CT data were processed using CTAn software (version 1.16.4.1, http://bruker-microct.com/products/downloads.htm#ctan) which was provided with the purchase of the Bruker X-ray micro-CT system. *: *p* < 0.05, ***: *p* < 0.01.

To evaluate the function of newly formed trabecular bone by local intramedullary injury plus daily PTH, screw implants were placed and the survival of the trabecular bone was assessed three weeks after implant placement (Fig. 4a). In the sham side where no implants were placed in the newly formed trabecular bone, very little bone remained in the medullary cavity, while periosteal bone was retained (Fig. 4b,c). On the other hand, a large amount of the trabecular bone survived in the medullary cavity with implant placement. The diaphyseal tibia was segmented into regions for further assessments combining the area on the outside of implants (both ends), the area in between the implants, and combing the area encompassing the implants themselves (Fig. 4d). The bone mass found at the implant site was significantly higher than both ends (Fig. 4e–h). The bone volume fraction between the implants was higher than those at the sham site or both ends; however, the difference did not reach statistical significance. Thus, newly generated trabecular bone by local intramedullary injury plus PTH was retained after the cessation of PTH treatment only with implant placement. The maturity of the trabecular bone retained around the implant was assessed using Masson–Goldner trichrome staining. The retained bone had relatively large osteocyte lacunae and showed uneven staining with an irregular texture, suggesting that the retained bone was immature and subject to active remodeling (Supplementary Figure S1).

## Discussion

Bone marrow disturbances by local intramedullary injury followed by PTH or ALN administration for 14 days induced substantial localized ossification in the diaphyseal medullary cavity. Micro-CT assessment showed no statistical differences in trabecular parameters except in trabecular number between the metaphysis and diaphysis for PTH treatment. However, significant differences were noted in bone volume fraction, trabecular thickness, and trabecular number between the metaphysis and diaphysis with ALN treatment. Therefore, the newly formed bone, in the diaphysis, essentially had a similar trabecular structure to the metaphysis with PTH but not with ALN treatment. Furthermore, the newly formed trabecular bone in the diaphysis was structurally different when comparing the PTH and ALN groups. The diaphyseal trabecular bone in the ALN group was less anisotropic than that in the PTH group. This difference is presumably attributed to a difference in the bone metabolic effects between PTH and alendronate; PTH promotes bone remodeling in favor of bone formation, while alendronate maintains bone mass by suppressing bone resorption. In this study, the local intramedullary injury was performed in the diaphysis so that the metaphyseal trabecular bone was not directly disturbed. Hence, the newly generated bone was isolated from and unlikely the extension of the existing metaphyseal trabecular bone. Indeed, the newly generated bone was sequestered from the metaphysis, existing very localized in the diaphysis, as depicted in the micro-CT images. A great amount of periosteal bone formation was also noted in the group with local intramedullary injury plus PTH or ALN. This induction of the periosteal bone formation was an interesting unintended result. During surgery, a full-thickness flap was raised to visualize drilling sites. This causes hematoma formation between the bone surface and periosteum which has been shown to be rich in mesenchymal cells from the bone marrow and periosteum^20^. These cells responded to PTH and ALN to form periosteal bone. If deemed unwanted, the periosteal bone formation could be minimized by modifying the resected flap design.

In rats, bone fracture prompts hemostasis, followed by host inflammation, capillary invasion, mesenchymal cell migration, callus formation, woven bone formation, and finally remodeling^21^. Within the callus, woven bone is typically generated via endochondral ossification. Thus, callus formation is essential for fracture healing. The intermittent administration of PTH has been shown to increase callus volume by increasing numbers of chondro- and osteo-progenitors at the bone fracture site in rats^22^, resulting in enhanced fracture healing^14^. While ALN does not enhance fracture healing, it also exerts no negative effect^23,24^. One might speculate that the repair of bone marrow disruption in this study might follow a similar course to fracture healing. In a rat model of fracture healing, calluses are typically observed at 2 weeks post-fracture^25,26^. However, in the current study, ossification in the medullary cavity was hardly found at 2 weeks in the VC group, suggesting that the generated diaphyseal trabecular bone by local intramedullary injury is not involved in endochondral-based fracture healing.

Mechanical bone marrow ablation is a procedure used to study biological events during bone marrow regeneration and intramembranous bone formation. A small access hole is typically made at the distal end of the metaphysis of long bone and the entire bone marrow is aspirated through the hole^27^. After the completion of the bone marrow aspiration, the medullary space responds by filling in with a blood clot, followed by capillary invasion, the migration of undifferentiated mesenchymal cells, the proliferation of osteoprogenitors, trabecular bone formation, sinusoid formation with hematopoietic tissue development, and finally the resorption of the trabecular bone. In the course of this regeneration process, the transient trabecular bone is formed via intramembranous ossification^28^ and nearly fills the entire medullary cavity^29^. This temporary event, bone marrow space filled with fine bone trabeculation, is necessary for the restoration of the sinusoid^30^. In other words, bone marrow will not be regenerated without transient trabecular bone formation. The mechanical disruption of bone marrow prompts the formation of transient trabecular bone although the bone marrow disturbance created in this study was on a much smaller scale than the entire bone marrow ablation referenced in the literature and our local intramedullary injury model involved the disruption of a local sinusoid system. Hence, it is unlikely that the newly formed trabecular bone in this study followed callus formation mode of healing and instead represented transient trabecular bone formed via intramembranous ossification. Accordingly, ossified tissue was hardly detected in the marrow space at the local intramedullary injury site in the VC group. This suggests that the sequential events of transient trabecular bone formation, the sinusoid reconstitution, and the trabecular bone resorption were completed by day-14 in this local injury model.

To better understand the molecular events behind the diaphyseal trabecular bone formation, an osteogenic RNA array was performed. Despite the dramatic bone formation, no osteogenic genes were significantly upregulated in the medullary cavity with local intramedullary injury plus PTH or ALN compared with local intramedullary injury plus VC. However, both PTH and ALN suppressed Ctsk and Col2a1 significantly, and trended the fibroblast growth factor (Fgf1) decrease. Ctsk and Fgf1 are important in the differentiation of osteoclasts and bone marrow mesenchymal stem cells, respectively^31,32^. Hence, the suppression of Ctsk supports the survival of trabecular bone in the marrow cavity. Overall, the regulation of osteogenic genes by local intramedullary injury plus PTH or ALN was not as robust as anticipated from the micro-CT findings and histological assessments. Location and timing may be a factor in these results. A better assessment of the bone marrow gene expression could be achieved by analyzing the precise portion of the bone marrow next to the local intramedullary injury site within a few days of the local intramedullary injury.

In bone marrow ablation, newly formed trabecular bone is a transient product and is destined to be resorbed for the restoration of the sinusoid. It was unknown if this transient trabecular bone could be maintained. In this study, we hypothesized that the newly formed trabecular bone has a function to respond to mechanical stimuli and that it can be maintained with implant placement. To test this hypothesis, screw implants were placed in the newly formed trabecular bone in the medullary cavity and bone responses were evaluated. For this experiment, the local intramedullary injury plus PTH was chosen over ALN. We took into consideration that the adherence of ALN to bone is irreversible and that the use of ALN is associated with the development of osteonecrosis of the jaw and atypical femoral fracture^33,34^. After 3 weeks post-implant/sham surgery, we found that the newly formed trabecular bone nearly disappeared without implants; however, a great amount of bone was retained with implants. Therefore, the transient trabecular bone is functionally adaptive and can be maintained with implant placement. To further study the effect of implants on the maturity of the surrounding trabecular bone, we visualized collagen fibers in the ossified connective tissue via Masson–Goldner staining. The retained trabecular bone around the implant showed a mosaic pattern of mature and less mature bone. This may suggest that the retained trabecular bone was under active bone remodeling. It was noted that the periosteal bone that was formed as a by-product of local intramedullary injury was not resorbed in the sham group at 3 weeks after the cessation of PTH although the diaphyseal trabecular bone in the medullary cavity disappeared without implants. This indicates that the fate of newly formed bone is quite different between the endo- and peri-osteal environments. This observation may support our proposal that the trabecular bone formed after bone marrow disturbance is not an endochondral-based callus but an intramembranous-based transient trabecular structure for the sinusoid restoration.

Osteoperiosteal decortication, also known as bone marrow penetration, is a surgical technique to expose the bone marrow mesenchymal cells to the extracortical environment^35^. In orthopedics, it is used to promote the union process of delayed- or non-union fractures^36^, while in dentistry, decortication is performed to induce periosteal bone formation^37^. Regardless of disciplines, decortication is not performed to induce bone formation in the medullary cavity. The findings of this study clearly show that bone marrow disruption by local intramedullary injury followed by PTH or ALN administration induced sizable trabecular bone formation locally, which is functional and can be maintained with implant placement. Hence, this method may be utilized to improve the osseointegration of hip arthroplasty, bone-anchored prostheses after a lower-limb amputation, and dental implants. Since patients who require hip arthroplasty or dental implants are typically seniors in age, young growing rats used in this study to investigate bone responses to injury may not be ideal. Therefore, findings could be validated in similar experiments using aged animals. Nonetheless, findings of this study can be translated to a similar clinical setting and can serve to advance our knowledge in the field of bone marrow regeneration. This method holds promise as a tissue engineering/regeneration strategy in medicine and dentistry.

## Methods

### Animals and local intramedullary injury

Male Sprague Dawley rats (n = 41, 6-week-old) were used. Rats were maintained at 22 °C in 12-h light/12-h dark cycles with ad libitum access to water and a standard rodent diet. One rat died during surgery due to anesthesia. The experimental protocol was approved by the Institutional Committee on Use and Care of Animals at Fukuoka Dental College (Members: Drs. Masumi Hidaka, Toshio Izumi, Tetsuichiro Inai, Tetsuya Nagashima, Masanori Tokumoto, Hiroshi Hayakawa, Hiromitsu Morita, Hiroyuki Machida, Youichi Kawano, Mitsutoki Hatta, Hiroshi Kajiya, and Mr. Hiroteru Wasai) and performed in compliance with the ARRIVE Guidelines. After a week of acclimation, rats underwent local intramedullary injury on their left tibia under general anesthesia with isoflurane inhalation. Fur was removed and the surgical site was disinfected with povidone-iodine. A longitudinal incision was made and the skin, muscle, and periosteum were carefully reflected to expose the bone surface. Five consecutive drilling holes were made in the diaphysis using a dental handpiece with ½ dental round bur to penetrate the bone marrow space through the cortex (Fig. 1a). The soft tissue over the intramedullary injury site was then closed by surgical staples. The right tibia served as an internal control. Sham surgery was performed in the right tibia wherein an incision was made, the cortex was exposed, and the soft tissue was closed without the creation of an injury.

### Injections and implants

Thirty-three rats were randomly divided into three groups (n = 11 per group), which received either recombinant human PTH (Bachem, Switzerland) at 20 μg/kg/d, ALN (Sigma-Aldrich, USA) at 0.4 mg/kg twice a week, or saline (100 μL/d) as vehicle control (VC) for 14 days (Fig. 1b). Injections were initiated at the time of local intramedullary injury. Doses were optimized based on our previous work and the relevant literature^18,38,39^. The remaining rats were in the fourth group (n = 7), where local intramedullary injury was performed in both tibiae followed by daily administration of PTH for two weeks. At day-14, rats were anesthetized and the soft tissues were reflected to expose the portion of the tibiae where the local intramedullary injury was previously created. Two screw implants (Ti–6Al–4V ELI alloy, Φ 1.4 × 3.0 mm, Jeil Medical, Korea) were then placed, approximately 5 mm apart, in the area where the local intramedullary injury was originally performed under copious irrigation (Fig. 4a). The implant had a machined smooth surface texture. No implants were placed in the right tibia. After implant placement, rats were maintained for 3 weeks without treatment to evaluate the effect of the implants on bone survival.

### Quantitative PCR array

To evaluate the effect of PTH and ALN on osteogenic gene expression in the bone marrow, a PCR Array (PAMM-026ZA, Qiagen, Germany) was performed. The PCR Array profiled the expression of 84 genes related to osteogenic differentiation. Four rats were randomly selected from each group. Rats were euthanized by carbon dioxide asphyxiation and both tibiae were dissected. The tibiae were transversely cut at both ends of the midshaft (between the crest of tibia and the tibia fibula junction (TFJ)) and the bone marrow of the resultant midshaft was flushed with cold trizol and homogenized. The cortex was not included in the homogenized sample. Total RNA was extracted from the sample using the phenol–chloroform protocol^40^. cDNA was synthesized from 1 μg total RNA using the RT2 first strand kit (Qiagen) and qPCR was performed. Data were analyzed using R (version 3.2.0). Fold changes for each comparison were calculated as 2^(-ΔΔCT). The PCR Array was performed in the microarray core facility.

### Microcomputed tomography (micro-CT)

Rats were euthanized by carbon dioxide asphyxiation at the designated time points. Tibiae were dissected, fixed in 4% formaldehyde for two days, and saved in 70% ethanol at 4 °C. Implants were gently unscrewed from the tibiae. The whole tibia was scanned at 18-μm voxel resolution with an energy level of 45 kV, an x-ray intensity of 500 μA with 460 ms exposure time, and a sample rotation of 0.5° rotation step for 180° using micro-CT (Skyscan-1176, Bruker, Billerica, MA, USA). All grayscale images were set to a threshold between 100 and 255 grayscale units. The metaphyseal trabecular bone of the proximal tibiae from 1.2 to 2.7 mm distal to the growth plate (100 slices) was analyzed to establish baseline bone response to the treatment. For the assessment of the injury sites, a region of interest (ROI) was defined as the tibial midshaft from 0.9 mm mesial to the most proximal drill hole to 0.9 mm distal to the most distant drill hole (approx. 550 slices). The same ROI was applied to the sham surgery site. The endocortical and periosteal areas were segmented by the semi-manual contouring method and analyzed using built-in CTAn software (version 1.16.4.1, Bruker). For the tibiae in which implants were placed for 3 weeks, the bone area was segmented based on the implant position (Fig. 4d). The cortex was excluded and detailed assessments for the remaining trabeculae were carried out. For the sham site on the contralateral bone, the above ROI was applied for the trabecular bone assessment.

### Serum chemistry

The systemic effect of local intramedullary injury plus PTH or ALN administrations on calcium homeostasis, bone resorption, and bone formation was assessed. Serum samples were prepared from blood collected at baseline (BL) and day-14, stored at − 80 °C until use. Blood collection was performed in the afternoon without overnight fast. Serum calcium, tartrate-resistant acid phosphatase isoform 5b (TRAcP5b), and osteocalcin levels were measured in accordance with the relevant manufacturers’ protocols using C7503 (Pointe Scientific, USA), RatTRAP ELISA (IDS, UK), and Rat Osteocalcin ELISA (Immutopics, USA) kits, respectively.

### Histology and histomorphometric analysis

After micro-CT scanning, tibiae were decalcified in ethylenediaminetetraacetic acid (14%, pH 7.5), processed for paraffin embedding, and sectioned at the level of the local intramedullary injury. Hematoxylin and eosin (HE) staining and Masson–Goldner trichrome staining (Merck, Darmstadt, Germany) were performed. Tartrate-resistant acid phosphatase (TRAP) staining was conducted to visualize osteoclasts using a commercial kit (Sigma-Aldrich, USA). Stained sections were photomicrographed and histomorphometrically analyzed using Image-Pro Premier (Media Cybernetics, USA).

### Statistical analysis

Statistical analyses were performed using the paired t-test for dependent data (between left and right tibiae; between BL and day-14; between the metaphysis and diaphysis) and an analysis of variance (ANOVA). Tukey’s test was used for multiple comparisons. All statistical analyses were conducted with SYSTAT (Systat Software, USA). An α-level of 0.05 was defined as statistically significant. Results are presented as the mean ± SEM.

## Supplementary Information

Supplementary Figure.

## Data Availability

The datasets generated and/or analysed during the current study are available from the corresponding author on reasonable request.
