# Supplementary material for: Intramedullary injury combined with osteoporosis therapeutics regulates targeted local osteogenesis
Source: Sci Rep. 2021 Jan 12;11:205. doi: 10.1038/s41598-020-80316-y (PMC7804436; doi:10.1038/s41598-020-80316-y)
Supplement: Supplementary file 1 — Supplementary Figure. [file 41598_2020_80316_MOESM1_ESM.pdf]

Intramedullary injury combined with osteoporosis therapeutics regulates targeted local osteogenesis.

Yoko Miyazaki-Asato, Kiyono Koi, Hiroki Fujimoto, Kae Kakura, Hirofumi Kido
Tsukasa Yanagi, Junro Yamashita

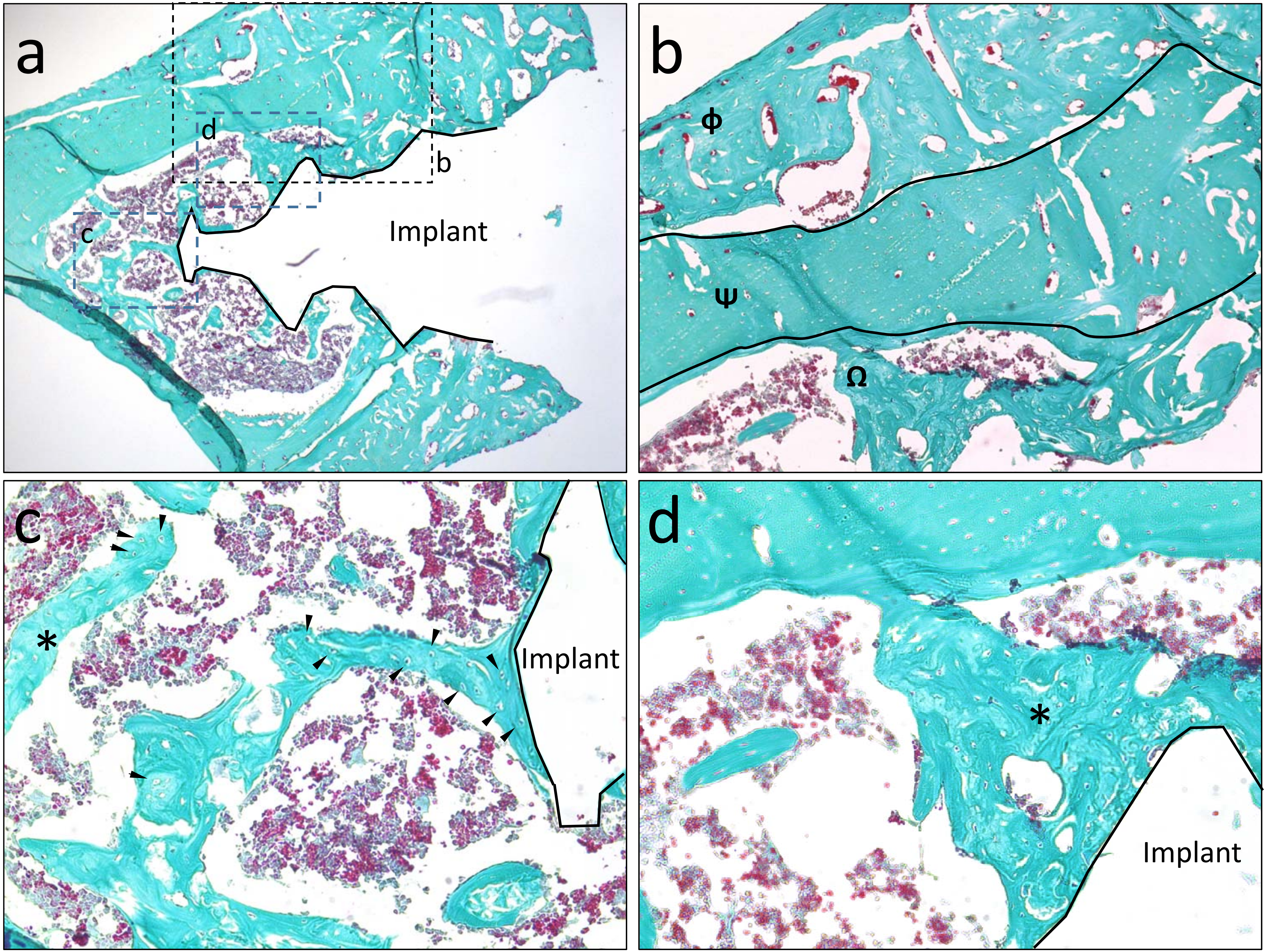
a
b
c
d
Implant
b
Φ
Ψ
Ω
c
*
Implant
d
*
Implant

Supplementary Figure S1: The retained trabecular bone around the implant was immature. Masson-Goldner trichrome staining was performed to visualize the maturity of bone around the implant. (a) A representative cross-sectional image of the diaphysis is shown. (b) The rectangular area “b” in image (a) is magnified. ϕ: periosteal reactive bone, Ψ: original cortex, Ω: endocortical reactive bone. (c) The rectangular area “c” in image (a) is magnified. Pale green-colored bone (*) is considered less matured, while darker green-colored bone is considered matured. Arrow heads indicate osteocyte lacunae. (d) The rectangular area “d” in image (a) is magnified.
